# Compressive Behavior of Composite Concrete Columns with Encased FRP Confined Concrete Cores

**DOI:** 10.3390/s19081792

**Published:** 2019-04-15

**Authors:** Xuxu Wang, Yujun Qi, Yunlou Sun, Zhijin Xie, Weiqing Liu

**Affiliations:** 1 College of Civil Engineering, Nanjing Tech University, Nanjing 211816, China; y928@njtech.edu.cn (X.W.); xzj1994@njtech.edu.cn (Z.X.); wqliu@njtech.edu.cn (W.L.); 2 College of Civil Engineering, Southeast University, Nanjing 210096, China; sunyl@seu.edu.cn

**Keywords:** fiber reinforced polymer (FRP), confined concrete, failure modes, average stress, ductility, Drucker–Prager model

## Abstract

A composite concrete column with encased fiber reinforced polymer (FRP) confined concrete cores (EFCCC) is proposed in this paper. The cross-sectional form of the EFCCC column is composed of several orderly arranged FRP confined concrete cores (FCCCs) surrounding a filled core concrete. This novel composite column has several advantages, such as higher compressive capacity, stronger FRP confinement, and ductile response. The compressive experiment is employed to investigate the compressive behavior of the EFCCC column with deferent parameters, such as outside concrete and stirrups. Test results show that the main failure mode of the EFCCC column with and without an outside concrete or stirrups is tensile fracture of the glass fiber reinforced polymer (GFRP) tubes. Compared to a reinforced concrete (RC) column, the strength and ductility of the EFCCC column was obviously improved by 20% and 500%, respectively. A finite element model (FEM) based on the Drucker–Prager (D-P) was developed that can accurately predict the axial compression behavior of the composite column with FRP confined concrete core. The predicted results obtained by using this FEM have excellent agreement with the experimental results.

## 1. Introduction

Currently, in regard to modern engineering structures, carrying capacity and deformation properties are in great demand [1]. In the past two decades, fiber reinforced polymer (FRP) composites have been widely applied in civil engineering construction, because of their several advantages, such as higher corrosion resistance, higher strength to weight ratio, and superior durability in aggressive environments [2]. 

Nowadays, FRP composites are often used to reinforce existing concrete members, such as beams and columns for building structures, and many researchers investigated the performance of reinforced concrete beams and columns with FRP [3,4,5,6,7,8]. Shaw et al. [5] conducted three-point bending tests on three small scale prestressed concrete (PC) beams that have been damaged then reinforced with carbon fiber reinforced polymer (CFRP) and glass fiber reinforced polymer (GFRP) externally bonded laminates. Results show externally bonded shear FRP can be used to regain and even exceed the shear capacity of the undamaged girder. Jiang et al. [9] made an experimental study of FRP-confined reinforced concrete (RC) columns involving different bonding conditions between FRP and concrete. It was found that variations in the bonding condition do not have a significant effect on the global response of the FRP-confined RC columns. However, slipping at the bond interface causes an adverse effect on the length of the plastic hinge zone. Related research [10] showed moisture ingress can severely deteriorate the long-term durability of FRP composite components. In addition, the temperature effect should not be overlooked, especially when the substrate is wood instead of concrete [10]. 

More recently, researchers have focused on the concrete-filled FRP tube (CFFT) with circular section for new structures in civil engineering construction. A number of investigation show that FRP confinement is very effective for circular columns [11,12]. The circular CFFT columns have high strength and excellent ductility due to the uniform confining pressure provided by FRP confinement. Xiao et al. [13] tested circular concrete stub columns with FRP composite jackets under axial compression. Results indicated that significant increase in strength and ductility of concrete can be achieved by FRP composite jackets. Wang et al. [14] conducted 16 FRP confined concrete stub column and found that by increasing the concrete strength, the peak load of column can be increased. Vincent et al. [15] investigated the effect of fiber angle on axial compressive behavior of circular FRP confined concrete. This investigation indicates that fibers used for FRP-confinement of concrete are most effective when aligned in the hoop direction, with fiber efficiency reducing significantly with an increase in fiber alignment with respect to the hoop direction. Guler et al. [16] reviewed design guidelines for CFFT with circular cross-sections, including many parameters, such as concrete strength, FRP tube thickness, fiber orientation, and slenderness ratio [17,18,19].

In fact, square and rectangular columns have a wide range of applications in building structure construction. It is now well recognized that square and rectangular tubes provide less effective confinement than circular tubes [12]. Therefore, for similar performance levels, square or rectangular FRP confined concrete columns require more confinement than circular FRP confined concrete columns, thus, requiring more FRP materials [20]. This could lead to a significant increase in construction costs. In the past decade, to solve the problem of FRP confinement being less effective for columns, researchers have conducted extensive investigations on FRP confined concrete columns with square or rectangular sections. Wu et al. [21,22] determined that the corner radius ratio is in direct proportion to the increase in confined concrete strength and that the confinement effectively increases the ductility of specimens composed of high-strength concrete. Ultimate strain increases with increasing corner radius [23]. The new square and rectangular CFFT system presented by Ozbakkaloglu [20] offers an extremely high confinement effectiveness that rivals circular CFFTs. These new square and rectangular CFFT systems were designed to enhance the effectiveness of square and rectangular FRP tubes in confining concrete. However, this new square and rectangular CFFT system was difficult to promote in actual engineering because this component is manufactured using a manual wet lay-up process. A novel steel–concrete–FRP–concrete (SCFC) [24] column effectively combines the merits of FRP, concrete, and steel and takes advantage of the interaction mechanisms among these layers. The SCFC column not only inherit the advantages of the FRP confined concrete core (FCCC) for the high-strength confinement of concrete but also further strengthen the bearing capacity and ductility of the confined column through the confinement of the steel tube. However, the steel in the SCFC column will be corroded in an aggressive environment as the outer layer of the column is wrapped by the steel tube, resulting in a serious decline in bearing capacity of the SCFC column. 

**Figure 1:**
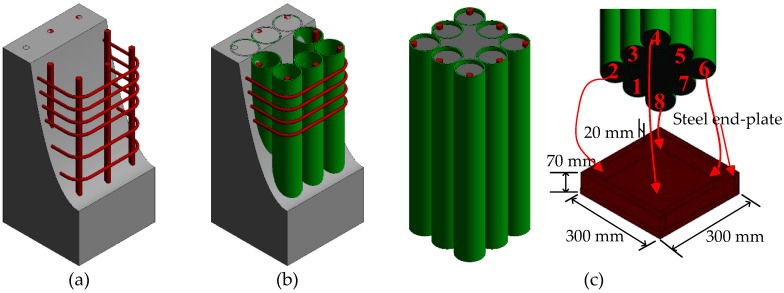
The constructions of all columns. (**a**) specimen T0; (**b**) specimen T8 and (**c**) specimen T8N.

In this paper, a novel hybrid column (shown in Figure 1) named encased FRP confined concrete cores (EFCCCs) column is proposed. The EFCCC column is composed of orderly arranged FRP confined concrete cores (FCCCs) and a filled core concrete. The interior FCCCs use circular cross-sections to achieve a higher confining pressure of the concrete. The hybrid column takes full advantage of the high compressive strength and good ductility of FCCCs. The EFCCC column is expected to solve the problem that FRP confinement is less effective for square or rectangular columns. A compressive experiment was conducted to understand the axial compression behaviors of EFCCC columns with and without outside concrete. In addition, the effects of stirrup spacing and outside concrete on the failure mode, load capacity, and ductility were also investigated. Furthermore, based on the Drucker–Prager (D-P) constitutive model, the finite element model (FEM) is adopted to predict the behavior of EFCCC columns. The D-P model was used to simulate the pseudo-ductile performance of the confined concrete inside the tubes.

## 2. Materials and Methods

### 2.1. Specimens Details

In this study, three types of composites columns (detailed in Table 1), as shown in Figure 1, were fabricated and tested. Two identical components for specimen T8N were poured to produce credible data. The production steps for the EFCCC column are as follows:

(1). The FCCCs were poured in the factory and transported to the laboratory;
(2). The stirrups were tied into a skeleton (for specimen T8) or the position of the prefabricated steel end-plate was fixed (for specimen T8N);
(3). The FCCCs were placed into the skeleton of the rebar (for specimen T8) or steel end-plates (for specimen T8N);
(4). The concrete was poured into the specified location. The detailed parameters, including the size (*b*×*h*), height (H) and tube quantity, are shown in Table 1.

The GFRP tubes were made from unsaturated resin and fiber fabricated by fiber winding technology, and all the tubes have identical heights and diameters of 550 mm and 77 mm, respectively.

**Figure 2:**
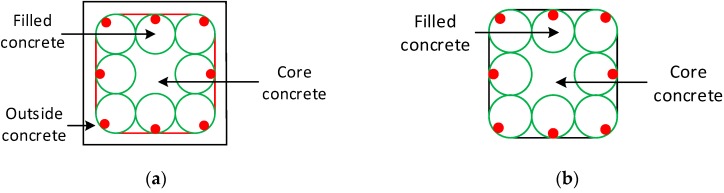
Cross-sections of the encased fiber reinforced polymer confined concrete cores (EFCCC) columns: (**a**) specimen T8 and (**b**) specimen T8N.

A total of four specimens were fabricated including one specimen T0 (Figure 1a), one specimen T8 (Figure 1b), and two specimens T8N (Figure 1c), all having the same height of 550 mm. The specimen T8 and the specimens T8N are two different types of EFCCC column. Specimen T0 is a reinforced concrete column and compared with the EFCCC columns. Specimen T8 contains 8 GFRP tubes (evenly distribute in the section), stirrups (evenly distribute at both ends of the column) and outside concrete. T8N only contains 8 GFRP tubes (evenly distribute in the section), no stirrups and no outside concrete. The longitudinal bars and stirrups were selected as Φ16 and ϕ8, respectively. Detailed parameters of these specimens are given in Table 1. Figure 2 shows the cross-section of the EFCCC columns and the names of the concretes with different properties in the cross-section.

### 2.2. Material Properties

Five GFRP tube samples with 3.5 mm thicknesses and 25 mm heights were tested in hoop tension in accordance with *ASTM D 2290-2012* [25] and *ASTM D 695-2010* [26]. Table 2 shows the material properties of the GFRP tubes.

Only C30 concrete was used to make the specimens in this study. The compressive strength of the concrete was measured from standard concrete cube tests (150 mm × 150 mm × 150 mm), according to the code for the design of concrete structures GB50010-2010 [27]; the experimental results are shown in Table 3.

### 2.3. Setup and Instrumentation

**Figure 3:**
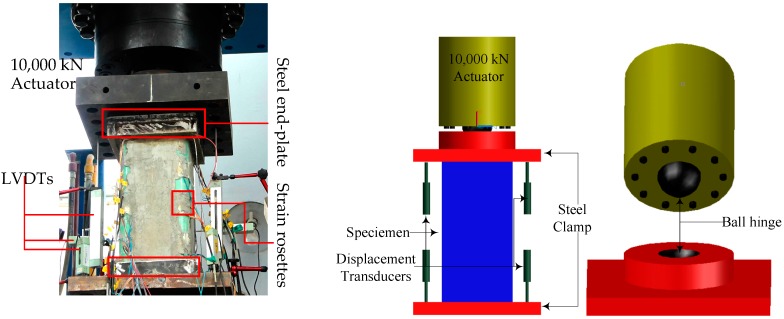
Test setup and instrumentation.

The experiments were conducted in the Structural Engineering Laboratory at Nanjing Tech University. The axial compressive loading was applied by a hydraulic actuator. The load capacity of the actuator was 10,000 kN. Each specimen was centered on the loading platform. Then, the whole section of each specimen was placed under compression produced by the loading machine, which was manually controlled at a displacement rate of 0.2 mm/min. Figure 3 shows the test setup and instrumentation. Last but not least, preloading was required before testing. To prevent local failures, the top and bottom surfaces of the tubes were pasted on 20 mm thick steel plates, and both ends of the tubes were reinforced by steel hoops for the T8N specimens. Bidirectional strain rosettes with gauge lengths of 10 mm were attached on the outer surfaces of the GFRP tubes at the mid-height of each tube. In addition, two linear variable differential transformers (LVDTs) with accuracies of 1 × 10^−3^ were installed at the top-height facing the load header to measure the axial deformation (Figure 3), while two other LVDTs were installed at bottom-height to record the settlement of the bearing plate. Measurements of strains, loads, and displacements were recorded simultaneously through a computer data logger. 

## 3. Results

### 3.1. Failure Modes

**Figure 4:**
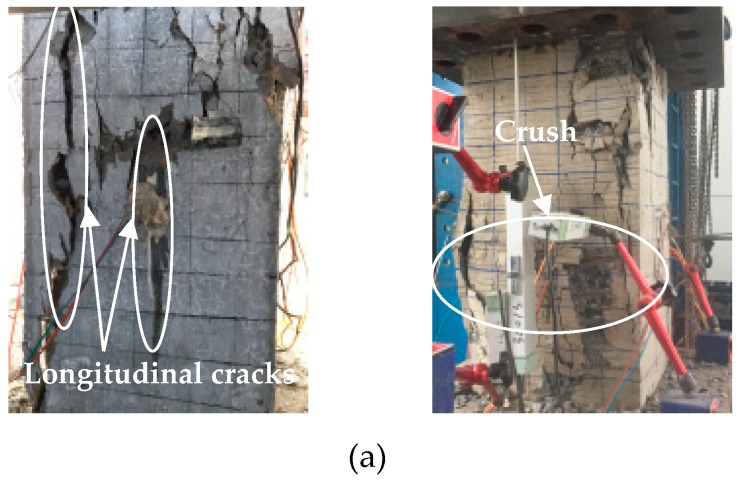

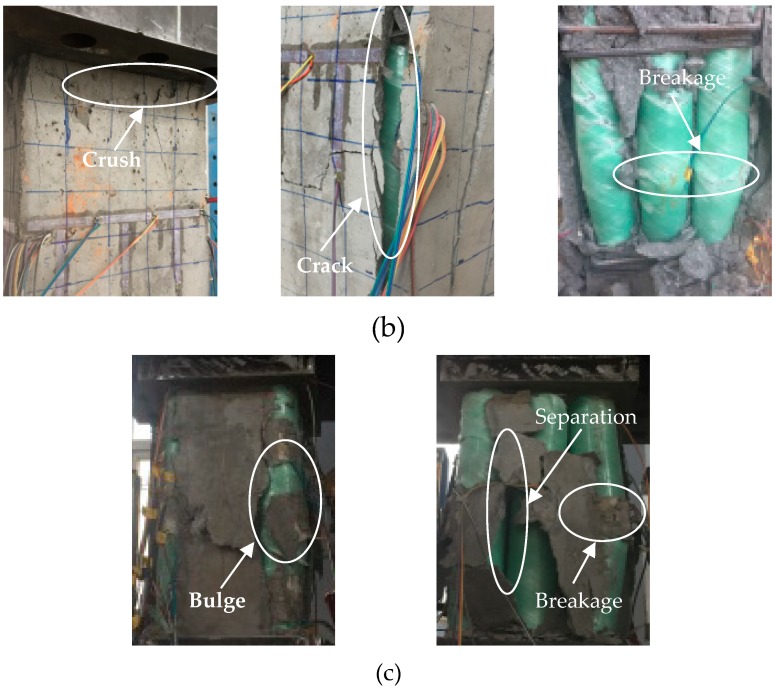
Failure modes of all the specimens: (**a**) specimen T0, (**b**) specimen T8 and (**c**) specimens T8N.

The failure modes of the specimens are shown in Figure 4. The initial damage in all specimens was concrete micro-cracking except for T8N specimens without outside concrete. The failure modes of all EFCCC columns were ruptures of the corner GFRP tubes after large area cracking occurred over the concrete surfaces. The compressive behaviors of the columns were significantly affected by the inner FCCCs and outside concrete.

Under larger loads, longitudinal cracks appeared in specimen T0 (shown in Figure 4a), which were roughly parallel to the direction of load. Then the concrete of protective layer peeled off, and finally, the concrete was crushed. For specimen T8 (shown in Figure 4b), a partial crushing first occurred near the loading plate, then a longitudinal crack occurred at the corner of the specimen due to an outward bulging of the GFRP tubes. The longitudinal cracks increased with increasing load. The GFRP tubes bulged continuously, which led to the outside concrete falling off. The load on the outside concrete transferred to the inner FCCCs and core concrete. The core concrete expanded outward under greater compressive load, which led an outward bending of GFRP tubes. Finally, the inner GFRP tubes ruptured at the tensile side. For specimens T8N (shown in Figure 4c), bulging of the GFRP tubes was initially observed, and then separation occurred between the GFRP tubes. Finally, like the specimen T8, the inner GFRP tubes ruptured by material breakage on the tension side.

### 3.2. Strength Analysis

The test results of all the columns, including the yielding load (*N_y_*), which can be obtained using the method presented in Section 4.1, peak load (*N_u_*), initial stiffness (*k*_1_), and hardening stiffness (*k*_2_), are shown in Table 4. For further quantification of the mechanical behavior of the EFCCC column some of the parameters, such as *γ* (*N_u_*/*N_y_*) and *μ* (*e_u_*/*e_y_*), are also summarized using the methods presented in Section 4.2 and Section 4.3, respectively, and the results are shown in Table 4; *γ* and *μ* represent the ratio of the peak load to the yielding load and the ratio of the corresponding displacements, respectively. Thus, *e_y_* and *e_u_* are the corresponding axial displacements. The axial stiffness of a column is defined as the slope of the load-displacement curve. The axial stiffness *k*_1_ is given by Equation (1), while *k*_2_ is given by Equation (2) through the method proposed by Feng [24].

(1) $$k_{1}=\frac{N_{y}}{e_{y}}$$

(2) $$k_{2}=\frac{N_{u}-N_{y}}{e_{u}-e_{y}}$$

Compared to specimen T0, which was manufactured by one-time monolithic pouring (*N_y_* = 2600 kN and *N_u_* = 2990 kN), the *N_y_* and *N_u_* of specimen T8 increased by 7.7% and 20.1%, respectively. *N_y_* increased by only 7.7% because the yielding load of a column is mainly determined by the cracking of the concrete. Due to the presence of eight internal FCCCs, the bearing capacity of specimen T8 was greatly improved.

The cross-sectional areas of the T8N specimens are different from the cross-sectional areas of specimen T0 and specimen T8. Therefore, to effectively compare the T8N specimens with specimen T0 and specimen T8, parameters *f_y_* and *f_u_* are introduced in this paper. The parameter *f_y_* is defined by Equation (3), and the parameter *f_u_* is given by Equation (4). The calculation results are shown Table 4.

(3) $$f_{y}=N_{y}/A$$

(4) $$f_{u}=N_{u}/A$$

The following variable definitions are used in the equations above:

*f_y_* and *f_u_* are the yielding stress and peak stress of a column, respectively; 

*N_y_* and *N_u_* are the yielding load and peak load of a column, respectively;

*A* is the cross-sectional area of a column (the cross-sectional areas of the T8N specimens include only the area of the eight FCCCs and the core concrete).

Compared to specimen T8 (*f_y_* = 36.49 MPa and *f_u_* = 46.79 MPa), the *f_y_* values of specimens T8N-1 and T8N-2 increased by 42.89% and 45.82%, respectively, and the *f_u_* values of specimens T8N-1 and T8N-2 increased by 44.56% and 48.72%, respectively. The *f_y_* values of T8 and T0 are almost the same due to invalid stress redistribution caused by the outside concrete while the effective stress redistribution induced larger *f_y_* values in T8N-1 and T8N-2. Compared to specimen T8 (*k*_1_ = 21.88 GPa and *k*_2_ = 0.7 GPa), the *k*_1_ values of specimens T8N-1 and T8N-2 decreased by 14.0% and 13.4%, respectively, and the *k*_2_ values of specimens T8N-1 and T8N-2 decreased by 2.9% and 10.0%, respectively. 

### 3.3. Load-Displacement Relationships

**Figure 5:**
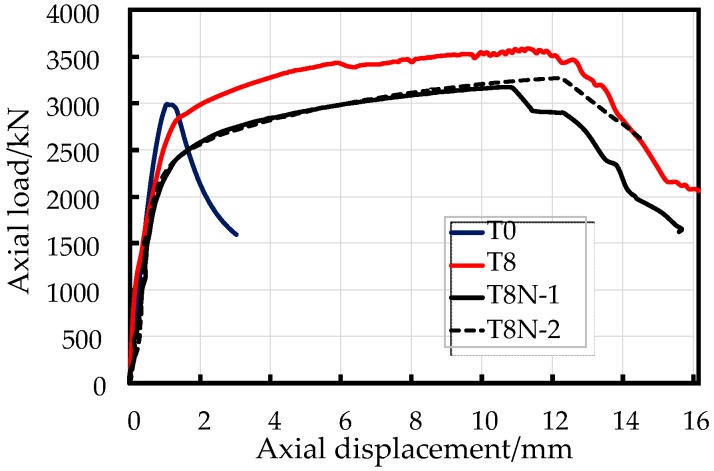
Axial load-displacement curves of specimens.

**Figure 6:**
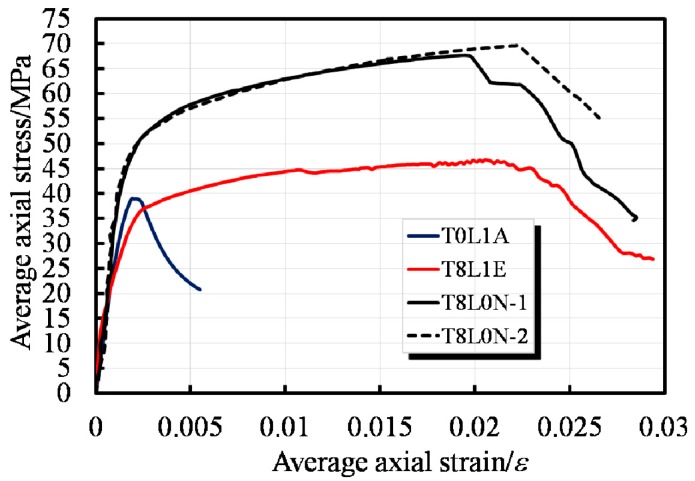
Average stress–strain curves of specimens.

Figure 5 shows the axial load-displacement curves for the EFCCC columns with RC column for comparison. In Figure 6 the axial load and axial displacement are normalized by the cross-sectional area of the column to eliminate the effect of the cross-sectional areas of the specimens, where the axial displacement was averaged from the readings of four LVDTs for each specimen, and the load was read from the force sensor on the universal loading machine.

Overall, the EFCCC columns showed similar load-displacement behaviors. The similar shapes of the axial load-displacement curves experienced monotonically ascending bilinear segments with large post peak deformations. However, compared to the RC columns, the yielding point occurred earlier, and the yield capacities were lower in the composite columns, which indicates that an unsatisfactory combination effect was achieved, namely, the original RC column integrity was weakened by the inner GFRP tubes. Moreover, the ultimate load in each EFCCC column was still higher than that of the common RC column because of the hardening process was observed (Figure 5).

It is worth noting that a large post peak deformation indicates predominant energy-saving because of the plasticity caused by eight FCCCs. With loading, a secondary ascending linear segment with a reduced slope appears after a short but smooth yielding stage. This finding suggests a significant stress redistribution effect after yielding of the EFCCC section. However, there is a short step reduction in the second-order stiffness during the hardening of specimen T8, while the second-order stiffness curves of the T8N specimens remain linear. This phenomenon can be attributed to a combination of cracking of the outside concrete and the smaller effect of the end stirrups due to the outward expansion of the composite tubes; therefore, the load-displacement curve of specimen T8 will gradually move closer to those of the T8N specimens.

Figure 5 and Figure 6 show that the axial peak load of the specimen T8 is higher than that of the T8N specimens because of the contribution of outside concrete. However, the average axial stress of the T8N specimens is much higher than that of the specimen T8, which was caused by the lower stress level of the unconfined outside concrete than that of the filled concrete and the core concrete.

### 3.4. Load–Strain Response

**Figure 7:**
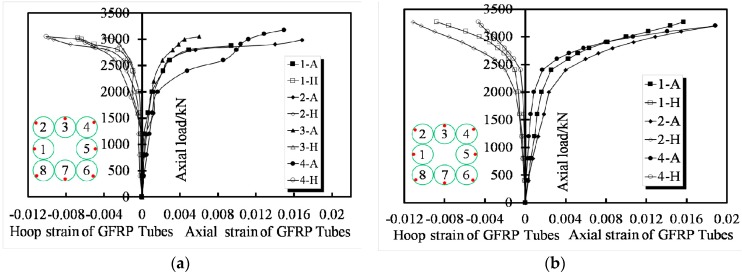
Typical load–strain response of GFRP tubes: (**a**) specimen T8N-1 and (**b**) specimen T8N-2.

A typical group of the load–strain curves obtained from the GFRP tubes of specimens T8N-1 and T8N-2 is shown in Figure 7a,b, respectively, and the data from just four tubes are revealed due to the symmetry of the specimens. The curves of the transverse strain and axial strain versus axial load are separately shown in the left and right parts of the figures. It is satisfactory that all curves exhibit an ascending bilinear shape and indicate significant enhancements in the strength and ductility of each specimen. Strain increases in the corner tube occurs, such as the No. 4 tube of T8N-1 and No .2 tube of T8N-2, after 2000 kN, and the rapidly increasing axial strain of the No .4 tube of T8N-1 shows that the stress distribution is dominated by the corner tube when the axial load is increased to 2500 kN. Large transverse displacements of the FCCCs due to the effective slenderness ratios were observed during the hardening of the specimens. As the slenderness ratio of the FCCC increased, the ultimate strength of the confined concrete decreased. The failure modes of the EFCCC columns were a tensile fracture of the GFRP tubes, and the direction of the fracture was determined by the fiber fabrication.

## 4. Theory Analysis

**Figure 8:**
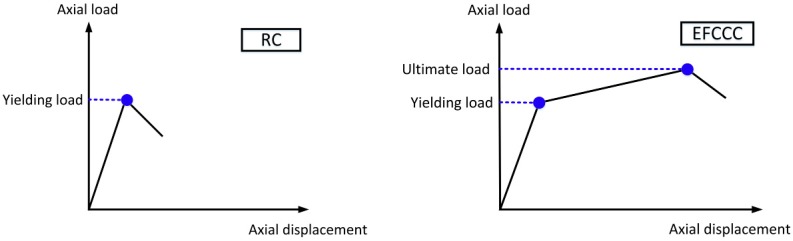
Mechanical behaviors of reinforced concrete (RC) and EFCCC.

What is different from the mechanical behavior of traditional confined concrete is that a decline curve with a certain residual strength dominated by the gradual failure of the FCCC can also be observed in Figure 5. Therefore, a tri-linear curve can be used to describe the compressive mechanism of the EFCCC columns under axial loading (Figure 8).

### 4.1. Yielding

**Figure 9:**
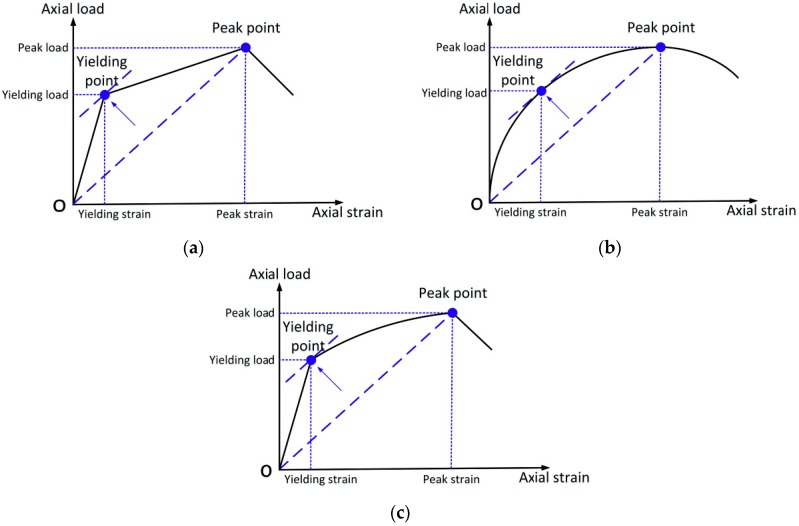
Method to determine the yielding point proposed by Feng [24]: (**a**) tri-linear curve; (**b**) curve without an obvious inflection point and (**c**) a traditional elastic–plastic curve.

The yielding state, which connects the linear stage and the subsequent hardening stage, is usually a point on the coordinate axis. To further analyze the yielding characteristics, the yielding load *N_y_* and its corresponding yielding displacement *e_y_* were obtained from the load–strain curve using Feng’s method [24]. Feng proposed a reasonable method to determine the yield point, wherein the yield point is the same as the slope of the tangent of the straight line connected by the peak point and the origin, as shown in Figure 9a. This method can be used to determine the yield point of a curve without an apparent yield point, as shown in Figure 9b. It is also feasible to apply this method to a traditional elastic-plastic curve, as shown in Figure 9c.

Using the method described above, the yielding load *N_y_* and yielding displacement *e_y_* of the different specimens were obtained and are shown in Table 4. Due to the weakening of the integrity of the original RC column by the inner GFRP tubes, the yielding load of specimen T8 was not greatly improved: the yield load of specimen T8 was only 7.7% greater than that of specimen T0.

### 4.2. Hardening Stage

After yielding, the concrete began to expand. Moreover, the expansion of the concrete in the eight FRP tubes caused the FRP tubes to have a passive confining force on the internal concrete. This passive confining force resists the expansion of the internal concrete, which resists the decrease in the stiffness of the specimen. Under the confinement of the FRP tube, the stiffness of the EFCCC column after yielding is usually stable at a roughly constant slope until the specimen reaches its peak. Therefore, this stage is defined as the hardening stage.

To evaluate the increase in load during the hardening stage, the ratio *γ* is introduced (called the load enhancement), which indicates the ratio of the peak load *N_u_* to the yielding load *N_y_.* This ratio is calculated by Equation (5).

(5) $$\gamma =\frac{N_{u}}{N_{y}}$$

The results of *γ* for different specimens are shown in Table 4.

### 4.3. Ductility

Ductility is an important characteristic of evaluating structural deformability. In this study, the method of ductility calculation that was defined in [28] was used. Namely, the maximum displacement was divided by the yield point displacement.

(6) $$\mu =\frac{e_{u}}{e_{y}}$$

where *μ* is the ductility ratio, *e_u_* corresponds to the maximum displacement, and *e_y_* represents the yielding displacement.

The maximum displacement *e_u_* of the EFCCC column is based on the displacement corresponding to the peak load. The ductility coefficient of each specimen is derived from Equation (6) and is listed in Table 4. The following conclusions can be drawn: (1) Compared with ordinary reinforced concrete components, EFCCC components can achieve higher ductility; (2) The ductility coefficient of specimen T8 is almost at the same level as those of the T8N specimens.

### 4.4. Theoretical Calculation

In existing models for FRP confined concrete, the confining pressure *f_l_* and ultimate strength of FCCC ($f_{cc}^{\prime }$) are determined using the following equations:

(7) $$f_{l}=\frac{2f_{frp}t_{f}}{d}$$

(8) $$\frac{f_{cc}^{\prime }}{f_{co}^{\prime }}=1+k_{1}\frac{f_{l}}{f_{co}^{\prime }}$$

where *d* is the inner diameter of the FCCC, *f_frp_* is the ultimate tensile rupture strength of the FRP, *t_f_* is the thickness of the FRP tube, $f_{co}^{\prime }$ is the compressive strength of unconfined concrete, $f_{cc}^{\prime }$ is the compressive strength of confined concrete and *k*_1_ is the factor of strength increase, which Teng suggested to set to 3.3 [29].

The influence of the effective slenderness ratio on reducing the ultimate strength of an FCCC can explained by the following formula [30]:

(9) $$\varphi =0.0013{\left(\frac{L}{D}\right)}^{2}-0.067(\frac{L}{D})+1.08$$

After using the calculation methods for the strengths of unconfined concrete, confined concrete, FRP tube, and steel [31], the strength of the EFCCC column can be computed by superposing the strengths of the EFCCC components, namely, the unconfined concrete, confined concrete, FRP tube, and longitudinal bars, as expressed in Equation (10) through Equation (13).

(10) $$N_{yb}=(A_{c1}+A_{c2}+A_{c3})f_{co}^{\prime }+A_{f}\sigma_{fa}+A_{s}f_{s}$$

(11) $$N_{yc}=(A_{c1}+A_{c2})f_{co}^{\prime }+A_{f}\sigma_{fa}+A_{s}f_{s}$$

(12) $$N_{ub}=A_{c1}f_{co}^{\prime }+A_{c2}f_{cc}^{\prime }+A_{f}\sigma_{fa}+A_{s}f_{s}$$

(13) $$N_{uc}=A_{c1}f_{cc}^{\prime }\varphi +A_{c2}f_{co}^{\prime }+A_{f}\sigma_{fa}+A_{s}f_{s}$$

The following variable definitions were used in the equations above:

*N_yb_* and *N_yc_* represent the yielding strength of specimen T8 and the T8N specimens, respectively;

*N_ub_* and *N_uc_* represent the ultimate strength of specimen T8 and the T8N specimens, respectively;

*A*_*c*1_, *A*_*c*2_ and *A*_*c*3_ represent the cross-sectional areas of concrete “Core concrete”, concrete “Filled concrete” and concrete “Outside concrete”, respectively, in Figure 2a,b;

*A_f_* and *A_s_* represent the cross-sectional areas of the FRP tube and longitudinal bars, respectively;

*σ_fa_* and *f_s_* represent the cross-sectional stress of the FRP tube and longitudinal bars, respectively.

Table 5 shows the comparisons between the experimental and theoretical results, where *N_y_* and *N_u_* are the experimental results and *N_y__T_* and *N_uT_* are the theoretical results. Note that “Avg” indicates the average value.

## 5. Numerical Simulation and Analysis

### 5.1. Description of the Finite Element Model (FEM)

The simulations of all the experimental tests all performed by using ANSYS, which is a commercial finite element program. Four-node shell41 elements, which are 3-D elements that have membrane (in-plane) stiffness but no bending (out-of-plane) stiffness, were used for the GFRP tubes, two-node link180 elements were used for the rebar, and eight-node solid65 elements that are capable of cracking (in three orthogonal directions), crushing, plastic deformation, and creep were used for the concrete.

Direct tests of the concrete, rebar, and GFRP tubes were conducted before the study. To accurately predict the behavior of the concrete, the actual stress–strain relationships of unconfined concrete and rebar were used in the finite element model. Moreover, a constitutive model that adaptively considers the surrounding pressure provided from the tubes is essential for the FEM. A critical review and assessment shows that the Drucker–Prager (D-P) plasticity model can successfully predict the behaviors of FRP-confined and other passively confined concrete [32,33,34]. The yield, nonrelated flow, and hardening rules considered in the D-P model can be determined by Equations (14)–(16).

(14) $$F=\sqrt{J_{2}}-\theta I_{1}-k$$

(15) $$d\varepsilon_{ij}^{p}=\lambda \frac{\partial G}{\partial \sigma_{ij}}$$

(16) $$k={\int \left\{\sigma \right\}}^{T}[M]\{d\varepsilon^{\mathrm{pl}}\}$$

**Figure 10:**
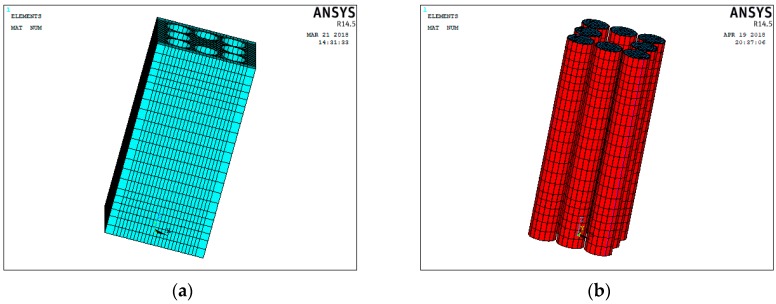
Finite element model of T8: (**a**) unconfined concrete and (**b**) fiber reinforced polymer confined concrete cores (FCCCs).

The cohesion and angle of the internal friction for concrete can be calculated by Equations (17) and (18).

(17) $$f_{\mathrm{co}}^{\prime }=\frac{2c\cos\phi }{1-\sin\phi }$$

(18) $$k_{1}=\frac{1+\sin\phi }{1-\sin\phi }$$

where $f_{\mathrm{co}}^{\prime }$ is the unconfined strength of concrete and *k*_1_ is the confined effectiveness factor, which Teng suggested should be set as 3.3 [29]. The established finite element model is shown in Figure 10.

### 5.2. Comparison of the Simulation Results

#### 5.2.1. Comparison of the Failure Mode and Load–Strain Behavior

**Figure 11:**
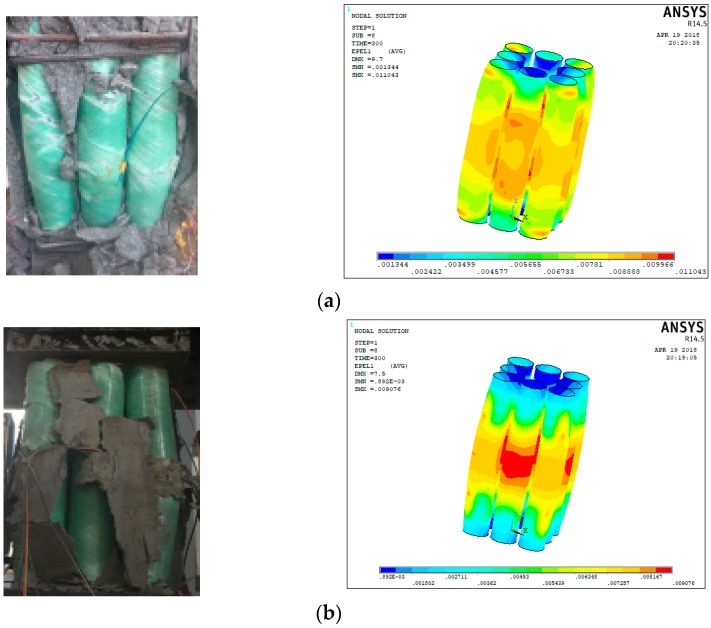
Comparisons of the failure mode of the tubes: (**a**) specimen T8 and (**b**) specimen T8N.

**Figure 12:**
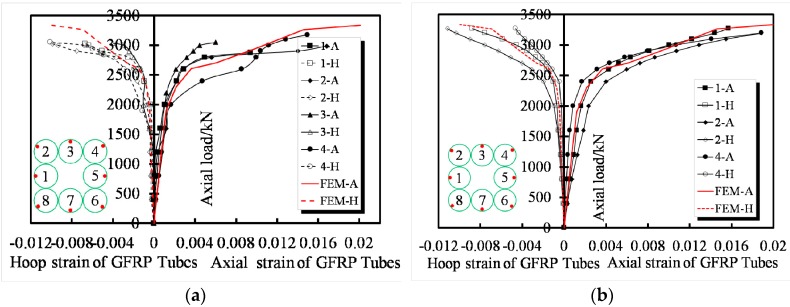
Comparisons of load–strain curves: (**a**) specimen T8N-1 and (**b**) specimen T8N-2.

The comparison of the failure modes of the tube obtained from experiments and finite element (FE) analysis is shown in Figure 11. The typical failure mode of the FRP tubes observed in the experiment is a serious outward bulging in the middle of the tubes. The load–strain curves of specimens T8 obtained from the FE analysis have good agreement with that obtained from experiments is shown in Figure 12. Since both ends of specimen T8 are restricted by the stirrups, the ends’ stress level of specimen T8 is significantly higher than that of T8N specimens without the stirrups. The high-stress level of T8N specimens is mainly concentrated in the middle of specimens.

#### 5.2.2. Comparison of Load-Displacement Behavior

**Figure 13:**
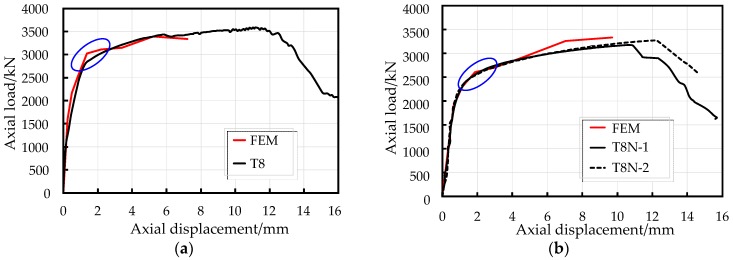
Comparisons of load-displacement curves: (**a**) specimen T8 and (**b**) specimen T8N.

The predicted yield load (*N_y_*) and ultimate axial compressive load (*N_u_*) were computed by the model proposed above. The load-displacement curves of EFCCC columns obtained from the FE analysis have good agreement with that obtained from experiments and is shown in Figure 13. However, the model did not accurately predict the ultimate displacement (*e_u_*) values of specimens T8 and T8N, which the model calculated to be 7.2 mm and 9.7 mm respectively; this inaccurate prediction was caused by the non-convergence of the outside unconfined concrete. The predicted results of the specimens are presented in Table 6. The average overestimation of *N_y_* by the proposed FEM was 6.0%. The mismatch between the marked blue circular area in Figure 13 shows that the FEM proposed above underestimated the stress redistribution between the outside concrete, filled concrete, and core concrete. The largest variation between the simulated and experimental results in *N_u_* was 222.5 kN, which occurred in specimen T8. No average error existed between simulated and experimental results in *N_u_*. 

The D-P model is often used to predict the behavior of FRP confined concrete. In this paper, the D-P model was employed to simulate behaviors of filled concrete and core concrete of the EFCCC column in this experiment. Predicted results are highly consistent with the experimental results in terms of failure modes and load-displacement curves. The development of this FEM based on the D-P model not only provides a basis for studying the mechanical behaviors of the EFCCC column but also provides a reference value for the prediction of the mechanical behavior of the composite columns with FRP confined concrete. 

## 6. Conclusions

A new type of composite column, which is referred to as a composite concrete column with encased FRP confined concrete cores (EFCCC), was introduced and tested under compressive loading. The failure modes, axial load-displacement relationships, and axial stress–strain responses were determined. The failure modes and mechanical characteristics were investigated. To determine the overall mechanical behavior of the novel composite columns, finite element models based on the D-P model were proposed. The corresponding major conclusions are summarized as follows:

(1). The reduced bearing capacity of the EFCCC column is dominated by the fractures of corner tubes, and the sequential failures of the other tubes. The mechanical behavior of the EFCCC column consists of three distinct stages, namely, the first linear stage, the yielding stage, and the hardening stage.
(2). Compared to specimen T8, the average yielding stress (*f_y_*) values of the T8N specimens increased by 42.89%, and the average peak stress (*f_u_*) values increased by 44.56%. These findings can be interpreted as the stress level of the unconfined outside concrete is much lower than that of the filled concrete and the core concrete. Compared to RC column, the ductility of the EFCCC column is increased by a factor of 5, and the ratio of the peak load to the yield load is also increased by a factor of 1.28–1.31, due to the effective stress redistribution caused by the built-in FCCCs.
(3). An analytical model that considers the effect of the slenderness ratio on the internal FCCCs of the column was developed to predict the axial behavior of the EFCCC column. The analytical predictions were generally in good agreement with the experimental results.
(4). A finite element model based on the D-P model was developed that considers nonrelated flow and hardening rules and has a satisfactory degree of agreement with the experimentally determined peak load, yielding load, and the peak load to yielding load ratio; however, the model did not accurately predict the yielding load and peak load of specimen T8N because the D-P model, which is the ideal plastic model, does not consider the cracking and damage of concrete. In future studies, many experiments with variable parameters should be conducted for the purpose of more meaningful results regarding EFCCC column.

To further understand the mechanical performance of EFCCC column and promote the utility of EFCCC column in building structure construction, the eccentric behaviors of EFCCC column should be investigated. The theoretical analyses and FEM based on the D-P model in this paper could be a reference to investigate the mechanical characteristics of EFCCC column under eccentric load.

## Figures and Tables

**Table 1:** Detailed parameters of the specimens.

Specimens	Size (*b*×*h*) (mm)	*H*^1^ (mm)	$f_{c}^{\prime }$^2^ (MPa)	*d*^3^ (mm)	*t*^4^ (mm)	Location of Stirrup	Location of Longitudinal Bars
T0	277×277	550	30	/	/	φ8@40 φ8@70	8Φ16
T8	277×277	550	30	77	3.5	φ8@40	8Φ16
T8N-1	231×231	550	30	77	3.5	/	8Φ16
T8N-2	231×231	550	30	77	3.5	/	8Φ16

^1^ Specimens height; ^2^ Concrete strength; ^3^ Outer diameter of GFRP tubes; ^4^ Wall thickness of GFRP tubes.

**Table 2:** Material properties of the GFRP tubes.

	Strength (MPa)	Ultimate Strain	Young’s Modulus (GPa)	Poisson’s Ratio
Hoop tension	(*f_frp_)* 365.10	(*ε_l_*) 0.030	(*E_l_*) 12.17	(*υ_l_*) 0.31
Axial compression	(*f_ac_*) 88.18	(*ε_ac_*) 0.031	(*E_ac_*) 2.82	(*υ_ac_*) 0.33

**Table 3:** Material parameters of concrete.

Cube Compressive Strength (MPa)	Axial Compressive Strength (MPa)	Axial Compressive Strain	Young’s Modulus (GPa)	Secant Modulus (GPa)
($f_{cu}^{\prime }$) 36.45	($f_{co}^{\prime }$) 28.80	(*ε_c_*) 0.00189	(*E_0_*) 25.38	(*E_p_*) 15.22

**Table 4:** Experimental and analytical results of the specimens.

	*N_y_* (kN)	*N_u_* (kN)	*A* (mm^2^)	*f_y_* (MPa)	*f_u_* (MPa)	*e_y_* (mm)	*e_u_* (mm)	*k*_1_ (GPa)	*k*_2_ (GPa)	*γ*	*μ*
T0	2600	2990	76729	33.86	38.97	0.72	1.04	36.11	3.75	1.03	1.44
T8	2800	3590	76729	36.49	46.79	1.28	11.28	21.88	0.70	1.28	8.81
T8N-1	2450	3178	46987	52.14	67.64	1.30	10.68	18.85	0.68	1.30	8.22
T8N-2	2500	3270	46987	53.21	69.59	1.32	12.16	18.94	0.63	1.31	9.21

**Table 5:** Theoretical calculation results.

	*N_y_* (kN)	*N_y_*_T_ (kN)	*N_y_*_T_/*N_y_*	*N_u_*(kN)	*N_u_*_T_ (kN)	*N_u_*_T_/*N_u_*
T8	2800	2989.56	1.07	3590	3723.11	1.18
T8N-1	2450	2351.13	0.96	3178	2910.94	1.03
T8N-2	2500	2351.13	0.94	3270	2910.94	1.01
Avg	-	-	0.99	-	-	1.07

**Table 6:** Comparison of experimental and analytical results.

	*N_y_* (kN)	*N_y_*_N_ (kN)	*N_y_*_N_/*N_y_*	*N_u_* (kN)	*N_u_*_N_ (kN)	*N_u_*_N_/*N_u_*	*γ*	*γ*_N_
T8	2800	3022.5	1.08	3590	3340.88	0.93	1.28	1.10
T8N-1	2450	2607.0	1.06	3178	3336.334	1.05	1.30	1.27
T8N-2	2500	2607.0	1.04	3270	3336.334	1.02	1.31	1.27
Avg	-	-	1.06	-	-	1	-	-
